# Scaling-up the Xpert MTB/RIF assay for the detection of tuberculosis and rifampicin resistance in India: An economic analysis

**DOI:** 10.1371/journal.pone.0184270

**Published:** 2017-09-07

**Authors:** Sunil Khaparde, Neeraj Raizada, Sreenivas Achuthan Nair, Claudia Denkinger, Kuldeep Singh Sachdeva, Chinnambedu Nainarappan Paramasivan, Virender Singh Salhotra, Anna Vassall, Anja van't Hoog

**Affiliations:** 1 Central TB Division, Government of India, New Delhi, India; 2 Foundation for Innovative New Diagnostics, New Delhi, India; 3 World Health Organization, Delhi, India; 4 Foundation for Innovative New Diagnostics, Geneva, Switzerland; 5 Additional DDG, Central TB Division, Ministry of Health and Family Welfare, New Delhi, India; 6 Department of Global Health, Amsterdam Institute of Global Health and Development, Academic Medical Center, Amsterdam, The Netherlands; 7 Department of Global Health and Development, London School of Hygiene and Tropical Medicine, London, United Kingdom; Institut de Pharmacologie et de Biologie Structurale, FRANCE

## Abstract

**Background:**

India is considering the scale-up of the Xpert MTB/RIF assay for detection of tuberculosis (TB) and rifampicin resistance. We conducted an economic analysis to estimate the costs of different strategies of Xpert implementation in India.

**Methods:**

Using a decision analytical model, we compared four diagnostic strategies for TB patients: (i) sputum smear microscopy (SSM) only; (ii) Xpert as a replacement for the rapid diagnostic test currently used for SSM-positive patients at risk of drug resistance (i.e. line probe assay (LPA)); (iii) Upfront Xpert testing for patients at risk of drug resistance; and (iv) Xpert as a replacement for SSM for all patients.

**Results:**

The total costs associated with diagnosis for 100,000 presumptive TB cases were: (i) US$ 619,042 for SSM-only; (ii) US$ 575,377 in the LPA replacement scenario; (iii) US$ 720,523 in the SSM replacement scenario; and (iv) US$ 1,639,643 in the Xpert-for-all scenario. Total cohort costs, including treatment costs, increased by 46% from the SSM-only to the Xpert-for-all strategy, largely due to the costs associated with second-line treatment of a higher number of rifampicin-resistant patients due to increased drug-resistant TB (DR-TB) case detection. The diagnostic costs for an estimated 7.64 million presumptive TB patients would comprise (i) 19%, (ii) 17%, (iii) 22% and (iv) 50% of the annual TB control budget. Mean total costs, expressed per DR-TB case initiated on treatment, were lowest in the Xpert-for-all scenario (US$ 11,099).

**Conclusions:**

The Xpert-for-all strategy would result in the greatest increase of TB and DR-TB case detection, but would also have the highest associated costs. The strategy of using Xpert only for patients at risk for DR-TB would be more affordable, but would miss DR-TB cases and the cost per true DR-TB case detected would be higher compared to the Xpert-for-all strategy. As such expanded Xpert strategy would require significant increased TB control budget to ensure that increased case detection is followed by appropriate care.

## Introduction

According to the 2015 World Health Organization (WHO) Global TB Report, India has the highest tuberculosis (TB) burden in the world. In 2015, an estimated 2.8 million people in India developed active TB, of which approximately 59% were diagnosed [1]. 28,876 patients received a laboratory-confirmed MDR-TB diagnosis, which is approximately one third of the estimated total number of MDR-TB cases [1]. Improving the detection of active TB and of MDR-TB is among the top priorities of the Revised National TB Control Program (RNTCP) [2], in line with the WHO End TB Strategy to improve patient-centered TB care and prevention.

The Xpert MTB/RIF assay (hereafter referred to as Xpert) for the diagnosis of TB and DR-TB offers great promise as a tool for TB control in India. The WHO-endorsed assay has greater sensitivity for TB detection than sputum smear microscopy (SSM) [3], high accuracy for the detection of rifampicin resistance [4], and provides a diagnosis of TB and MDR-TB from a single sample within 2 hours. Furthermore, a previous economic evaluation found Xpert to be cost-effective as a replacement for SSM in India [5], with the benefit of increased case detection. A multisite, phased implementation study conducted to collect evidence for the scale-up of the Xpert assay for TB and MDR-TB diagnosis in India found that the proportion of bacteriologically-confirmed TB cases identified, out of the presumptive TB cases tested, increased by 33% (95% CI 16–52%), while the detection of all patients diagnosed with bacteriologically-confirmed or clinically-diagnosed pulmonary TB, combined, increased by 11% (95% CI 3–21%) compared to SSM [6,7].

Although Xpert has been shown to be easy to implement, and is potentially cost-effective and successful in increasing the detection of TB and MDR-TB in India [6,7], Xpert access and uptake in India have been hindered by issues related to cartridge cost, procurement and supply chain management, a lack of engagement of the private sector (the first point of contact with the healthcare system for about 70% of patients in India), and a major national TB control program (NTP) financial deficit [8,9]. A country-wide implementation of Xpert would require a considerable increase in the NTP budget [10], and the projected, increased costs for TB diagnosis and treatment of additional, identified TB and MDR-TB cases through Xpert scale-up may well exceed the available budget and compete with other cost-effective health interventions [11]. For these reasons, it is critical to explore the benefits and trade-offs of different strategies to Xpert implementation in order to inform decisions about the scale-up of the Xpert assay in India. We conducted an economic analysis to determine the per-patient and overall costs of diagnosing and treating TB and rifampicin-resistant TB given different diagnostic scenarios.

## Methods

### Overview

We developed a decision analytical model to simulate a cohort of patients who report to public health facilities in India with symptoms or signs suggestive of TB and require diagnostic testing for pulmonary TB [7]. We compared four different scenarios that consider different combinations of diagnostic tests and the target population, or sub-population, for each test. True and false positive (TP/FP) and true and false negative (TN/FN) cases were estimated for all scenarios, assuming that the tests perform with reported sensitivity and specificity (Table 1) [3,4,12,13]. We estimated the total costs (in 2013 US dollars) of diagnosing and treating TB and rifampicin-resistant TB for the cohort, as well as average costs per presumptive TB patient tested, per true TB case and per rifampicin-resistant TB case detected and initiated on treatment. The primary analysis was conducted from the perspective of a public, TB health care provider. We also estimated the impact of the total costs of each scenario on the national budget for TB control [1].

**Table 1:** Model parameters.

Parameter	Point estimate	Range for deterministic sensitivity analysis		
		Low	High	Source
***Prevalence among simulated cohort***				
Proportion of patients known to have tuberculosis who require drug resistance testing ("MDR-suspect")	0.0245	0.000	0.0254	Sachdeva et al. 2014 [7]
Proportion of presumptive TB patients previously treated for TB	0.168	0.019	0.3522	Sachdeva et al. 2014 [7]; average and range observed across sites
Prevalence of true PTB among new presumptive TB patients	0.15	0.08	0.23	Assumption
Prevalence of true PTB among previously treated presumptive TB patients	0.266	0.183	0.3982	Assumption
Prevalence of RIF resistance among new patients	0.022	0.019	0.0260	WHO report 2014 [1]
Prevalence of RIF resistance among previously treated patients and DR-suspects	0.150	0.110	0.1900	WHO report 2014 [1]
***Diagnostic test accuracy***				
Sensitivity of Xpert MTB/RIF for diagnosing TB	0.89	0.85	0.92	Steingart et al. 2014 [4]
Specificity of Xpert MTB/RIF for diagnosing TB	0.99	0.98	0.99	Steingart et al. 2014 [4]
Sensitivity of Xpert MTB/RIF for Rif detection	0.95	0.90	0.97	Steingart et al. 2014 [4]
Specificity of Xpert MTB/RIF for Rif detection	0.98	0.97	0.99	Steingart et al. 2014 [4]
Sensitivity of sputum smear microscopy (ZN)	0.62	0.44	0.79	Steingart et al. 2006 [12]
Specificity of sputum smear microscopy (ZN)	0.98	0.96	1.00	Steingart et al. 2006 [12]
Sensitivity of LPA in detecting rifampicin resistance	0.99	0.96	1.00	Bwanga et al. 2009 [26]
Specificity of LPA in detecting rifampicin resistance	0.99	0.98	1.00	Bwanga et al. 2009 [26]
Sensitivity of clinical diagnosis in case found negative sputum smear microscopy	0.160	0.10	0.22	Vassall 2011[5]
Alternative source for Sensitivity of clinical diagnosis in case found negative sputum smear microscopy	0.610			Walusimbi 2013 [24]
Specificity of clinical diagnosis in case found negative sputum smear microscopy	0.942	0.93	0.97	Vassall 2011[5]
Alternative source for specificity of clinical diagnosis in case found negative sputum smear microscopy	0.690			Walusimbi 2013 [23]
Proportion of sputum smear negative patients getting CXR	0.037	0	1	Vassall 2011[5]
Ratio of proportion of Xpert-negative patients who get CXR, compared to proportion of smear-negative	0.5	0	1	Assumption
Proportion of Xpert positive patients who are smear negative	0.36	0.355	0.368	Sachdeva et al. 2014 [7]
***Diagnostic test unit costs (in US$ 2013)***				
Cost of sputum smear microscopy (per patient tested)	0.83	0.60	1.10	Microcosting study. Rupert et al. 2017 [15]
Cost of Xpert testing, per patient tested	13.17	12.06	14.72	Microcosting study. Rupert et al. 2017 [15]
Cost of liquid culture, per patient tested	13.42	10.51	16.21	Microcosting study. Rupert et al. 2017 [15]
Cost of line probe assay for DST, per patient tested (1)	21.34	19.50	23.07	Microcosting study. Rupert et al. 2017 [15]; adjusted for 2.2% error rate
Cost of antibiotic trial in clinical diagnosis	3.86	3.86	3.86	Vassall 2011[5]
Cost of CXR	4.0	2.98	5.00	WHO planning and budgeting tool [17]
Cost of clinical diagnosis = cost of antibiotic trial in all patients + cost of CXR multiplied by the proportion of patients getting CXR				
***Treatment costs***				
Full first-line regimen (2RH/4EHRZ)	$148	$93	$188	S1
Full first-line retreatment regimen	$189	$185	$305	S1
Second-line standard regimen, average for 24 months	$5,812	$4,204	$7,421	S1; Fitzpatrick 2012 [21]
Number of weeks on first-line treatment until confirmatory results of LPA are returned in new Xpert RIF positive patients	2.5			Assumption
***Loss to follow-up before treatment initiation (3)***				
Proportion of sputum smear positive patients not starting treatment in the baseline	0.092	0	0.20	Sachdeva et al. 2014 [7]
Proportion of bacteriologically positive TB patients not starting treatment in the intervention phase	0.099	0	0.20	Sachdeva et al. 2014 [7]
Proportion clinically diagnosed TB patients not starting treatment	0			Assumption
Additional initial default in DR-TB patients	0.10	0	0.2	Sachdeva et al. 2014 [7]

### Population

The simulated population represented adult patients requiring diagnostic testing for TB, rifampicin-resistant TB or both. In the primary analysis, the cohort characteristics were stipulated as observed in the implementation study [7], except for the prevalence of bacteriologically-confirmed (culture positive) pulmonary TB, which was adapted to the national average (15% in new patients and 27% in patients previously treated for TB) in order to make the conclusion more attributable to the national program and not just to the areas included in the implementation study. In the implementation study [7], the proportion of presumptive TB patients previously treated for TB, out of all patients with presumptive TB was 16.8% (11,922/70,556) and the prevalence of rifampicin resistance was 5.6% in new and 24.6% in previously treated TB patients. Two percent of the cohort comprised TB patients requiring drug resistance testing because of treatment failure (DR-TB suspects). HIV-infection was not considered due to low prevalence of HIV in India. See Supplement S1 File for further details.

### Diagnostic scenarios

#### Scenario 1. Baseline: Sputum smear microscopy (SSM) only

The baseline scenario represents the recommended practice in India [14], with SSM being the primary diagnostic tool for screening of all presumptive TB patients followed by line probe assay (LPA) screening of patients at increased risk for MDR-TB as recommended by RNTCP. Briefly, SSM was performed on two samples from all presumptive TB patients. Patients with ≥1 positive SSM were considered a TB case and required TB treatment. SSM negative patients were clinically evaluated following the RNTCP algorithm (Fig A of S1 File). For patients with two negative smears, the recommendation consisted of 10–14 days of antibiotics followed by two repeat SSM examinations if cough persisted. If these SSM results were negative, TB treatment may be initiated based on a chest x-ray (CXR) suggestive of TB [7]. In the primary analysis we attempted to closely reflect actual practice, and thus assumed that all patients received an antibiotic trial, though only a portion of patients received a CXR [4,13]. In a sensitivity analysis, the NTP’s newly-proposed algorithm that includes a CXR for all SSM negative patients with presumptive TB (in locations where Xpert is not accessible) was explored.

To diagnose rifampicin resistance, the sputum of SSM positive TB patients at increased risk for MDR was tested by LPA. In the model, in all scenarios, patients at increased risk for MDR included TB patients who failed first-line treatment (DR-TB suspects) as well as patients with a history of TB treatment in the past. This was chosen to stay close to the RCNTP guidelines which define high-risk TB cases as those TB cases with previous history of anti TB treatment, TB cases on treatment with positive sputum smear result at any follow up smear examination, diagnosed TB cases with HIV-co-infection and all pulmonary TB cases who are contacts of a known MDR TB case [9]. Patients with SSM negative (i.e. clinically diagnosed) TB were not further tested for drug resistance. Patients with a rifampicin-resistant LPA result were eligible for second-line TB treatment, according to RNTCP guidelines [9]. Patients newly diagnosed with TB were started the standard first-line, 6-month treatment regimen (Cat I), and those previously treated were started on a first-line, 8-month re-treatment regimen (Cat II).

#### Scenario 2. Xpert MTB/RIF as a replacement for LPA testing

This scenario was identical to the baseline scenario, except that the Xpert assay replaced LPA for DST following SSM, and thus was only applied to SSM-positive TB patients at increased risk for MDR.

#### Scenario 3. Upfront Xpert MTB/RIF testing for presumptive TB patients at increased risk of MDR

In the third scenario, presumptive TB patients at increased risk for MDR-TB (as defined in scenario 1) received upfront Xpert testing, rather than SSM. New patients were tested with SSM as in the baseline scenario. Thus, if new patients were diagnosed with pulmonary TB, they were not subjected to drug resistance testing.

#### Scenario 4. Upfront Xpert MTB/RIF for all presumptive TB patients

In the upfront Xpert-for-all scenario, all presumptive TB patients were tested up-front by Xpert, instead of SSM, regardless of previous history of TB. This scenario is in line with WHO recommendations [5]. If positive for MTB by Xpert testing, the patient was considered a TB case and required TB treatment. For presumptive TB cases with a negative Xpert result for MTB, we assumed that with significantly higher detection rates with Xpert, the proportion of patients subjected to the clinical diagnostic process would be half of those undergoing the clinical diagnostic process following SSM (the baseline scenario) [7]. In the sensitivity analysis, we explored the possibility of fewer patients being subjected to clinical diagnosis after a negative Xpert result.

After a rifampicin-sensitive Xpert result, a first-line treatment regimen was started (Cat I or II, according to retreatment status). In case of a rifampicin-resistant result for previously treated TB cases, second-line treatment was indicated without further diagnostic confirmation. In new cases, a sputum sample for confirmatory DST by LPA was required. Xpert MTB-positive, SSM-negative patients, assumed to be 36% [7], for which direct LPA cannot be done on the patient specimen, required a culture followed by an LPA on the isolate. Second-line treatment was started and changed to first-line treatment if MDR was not confirmed by LPA.

### Costs of diagnostic testing

An observational micro-costing study (or ‘bottom-up’ approach) was conducted at seven study sites to determine the cost of each bacteriological test (SSM, Xpert, LPA, culture, DST) per patient tested. The results are reported elsewhere [15]. The costs of clinical diagnosis were taken from earlier studies conducted in India [4]. The costs of TB treatment regimens were obtained by applying country-specific cost estimates, including cost of drugs, as well as tests and visits required for treatment monitoring [16–24] (Table 1; A detailed example is available in the Supplement S1 File).

### Outcome measures

The outcomes calculated by the model were the number of TB cases detected and initiated on TB treatment, divided into true positive and false positive cases as determined by phenotypic methods. Similarly, MDR cases detected and initiated on treatment were divided in true positive and false positive cases. For the primary analysis, estimates for primary loss to follow-up between bacteriological TB diagnosis and treatment initiation were taken from the two arms of the Xpert implementation study (9.2 and 9.9% in the baseline and Xpert-for-all scenario, respectively). Loss to follow-up for DR-TB cases before treatment initiation was also estimated to be 10% based on the previous implementation study [7]. To calculate the impact of the rollout of different scenarios to the whole country on the national budget for TB control, we assumed 610 presumptive TB patients were tested per 100,000 people per year, as observed in the implementation study [7]. Assuming a population of 1,252 million and a TB control budget of 252 million US$ [1], 7,637,200 presumptive TB patients would be tested per year.

### Sensitivity analyses

Deterministic sensitivity analyses were conducted to explore how assumptions and variations in the parameter estimates affected the total costs of different scenarios, including variation in the epidemiological parameters, assumptions about test accuracy, as well as the costs of diagnostic tests and treatment. Additionally, the assumptions about the use and interpretation (i.e. sensitivity and specificity) of CXR were explored in more detail, as little data is available regarding the proportion of patients receiving CXR in different diagnostic algorithms, despite the fact that the availability of CXR results could substantially affect clinicians’ decisions regarding empiric therapy and affect diagnostic costs. In the primary analysis, all patients with negative bacteriology (SSM) results were clinically evaluated, though only 4% of these patients received a CXR as part of their TB clinical assessment. This estimate was based on previous observations [4, 13], and resulted in low sensitivity and high specificity of the clinical diagnostic algorithm. In a sensitivity analysis, we explored the effect of different clinical diagnostic scenarios on cost and case detection in patients negative for MTB by bacteriological tests. We also explored the effect of increasing the proportion of these patients receiving a CXR to 100%, which would increase the sensitivity and lower the specificity [10].

## Ethics

Ethical approval was not obtained for this study, as only secondary data were used in these analyses.

## Results

### Cohort of 100,000 persons

The proportion of true pulmonary TB cases diagnosed and initiated on treatment among presumptive TB patients was 62% in both the SSM-only and LPA replacement scenarios, 67% in the SSM replacement scenario, and 81% in the Xpert-for-all scenario (Table 2). The total number of patients on TB treatment, in a cohort of 100,000 persons, was highest in the Xpert-for-all scenario (16,076), but the proportion of false-positive TB diagnoses was lowest in this scenario. Detection of true rifampicin-resistant TB cases was highest (73%) in the Xpert-for-all scenario, compared to SSM-only (48%), Xpert as an LPA replacement (46%) and Xpert as a SSM replacement (59%).

**Table 2:** TB and rifampicin resistant-TB cases detected and initiated on treatment by each of the 4 diagnostic strategies, in a cohort 100,000 persons.

	TB case detection				DR-TB case detection				
	True TB cases detected and initiated on treatment (TP)		False TB cases detected and initiated on treatment (FP)	Total number on TB treatment (TP+FP)	True DR-TB cases detected and initiated on treatment (TP)		False DR-TB cases detected and initiated on treatment (FP)	% of all persons with DR-TB diagnosis	Total number on DR-TB treatment (TP+FP)
Diagnostic Strategy*	n	(%**)	n	n	n	(%)	n	%	n
**1. SSM Only**	10,188	62%	5,365	15,553	620	48%	36	5%	655
**2. Xpert MTB/RIF as a replacement for LPA testing**	10,188	62%	5,365	15,553	595	46%	71	11%	665
**3. Xpert MTB/RIF as a replacement for SSM for patients with previous TB history**	11,016	67%	4,969	15,985	762	59%	99	12%	861
**4. Xpert MTB/RIF for all patients**	13,380	81%	2,697	16,076	945	73%	101	10%	1,046

TP = true positive; DR-TB = drug-resistant TB, i.e. rifampicin-resistant; FP = false positive; SSM = sputum smear microscopy

*Scenario 1: Perform SSM. If SSM positive and patient has been previously treated for TB, use LPA. If SSM negative and patient has been previously treated for TB, perform culture, LPA and/or DST.

Scenario 2: Perform SSM. If SSM positive and patient has been previously treated for TB, use Xpert in place of LPA. If SSM negative and patient has been previously treated for TB, perform culture, LPA and/or DST.

Scenario 3: Perform SSM only for patients not at DR-TB risk. Perform Xpert MTB/RIF for patient has been previously treated for TB.

Scenario 4: Perform Xpert MTB/RIF for all patients, regardless of DR-TB risk.

**Out of 16,492 true pulmonary TB cases among presumptive TB patients and 1,288 rifampicin resistant cases in the cohort

The diagnostic costs for the diagnosis of TB and rifampicin-resistant TB in a cohort of 100,000 persons (Table 3) were: US$ 619,042 in the SSM-only scenario, US$ 575,377 in the LPA replacement scenario, US$ 720,523 in the SSM replacement scenario, and US$ 1,639,643 in the Xpert-for-all scenario. In all scenarios, diagnostic costs were a minor proportion (8–16%) of total cohort costs, of which treatment costs were the largest component. Total cohort costs increased by 46% in the Xpert-for-all scenario and 19% in the LPA replacement scenario compared to SSM-only. This increase is largely due to the increased costs associated with second-line treatment of the additional rifampicin-resistant TB patients identified upon up-front Xpert testing. The incremental total cohort cost per true TB case initiated on treatment, compared to baseline was US$ 1040 for scenario 4, and $ 1614 for scenario 3 (Table 4).

**Table 3:** Total diagnostic costs and treatment costs to test 100,000 patients, for 4 diagnostic strategies (Cost in US$2013).

	Total diagnostic costs**	% of Total cohort costs	Patients on TB treatment (TP+FP), any regimen	Patients on DR-TB treatment (TP+FP)	Costs for 1st line treatment	Costs for 2nd line treatment	Total Treatment costs	% of Total cohort costs	Total cohort costs	Incremental total cohort costs compared to baseline	
Diagnostic Strategy*	US$ 2013		n	n	US$ 2013	US$ 2013	US$ 2013		US$ 2013	US$ 2013	%
**1. Baseline: SSM. Clinical diagnosis. If prev treated, LPA if SSM+ or Culture/LPA/DST if SSM negative**.	619,042	9%	15,553	655	2,747,408	3,807,122	6,554,530	91%	7,173,573		
**2. Baseline: SSM. Clinical diagnosis. If prev treated, Xp to diagnose Rif Res if SSM+**	575,377	8%	15,553	665	2,745,435	3,867,745	6,613,180	92%	7,188,556	14,984	0.2%
**3. Xpert MTB/RIF for presumptive TB cases with prev TB history. SSM for new patients**.	720,523	8%	15,985	861	2,782,695	5,006,646	7,789,340	92%	8,509,863	1,336,290	19%
**4. Xpert MTB/RIF for all patients**	1,639,643	16%	16,076	1,046	2,771,662	6,081,091	8,852,753	84%	10,492,396	3,318,823	46%

TP = true positive; DR-TB = drug-resistant TB, i.e. rifampicin-resistant; FP = false positive; SSM = sputum smear microscopy

*Scenario 1: Perform SSM. If SSM positive and patient has been previously treated for TB, use LPA. If SSM negative and patient has been previously treated for TB, perform culture, LPA and/or DST.

Scenario 2: Perform SSM. If SSM positive and patient has been previously treated for TB, use Xpert in place of LPA. If SSM negative and patient has been previously treated for TB, perform culture, LPA and/or DST.

Scenario 3: Perform SSM only for patients not at DR-TB risk. Perform Xpert MTB/RIF for patient has been previously treated for TB.

Scenario 4: Perform Xpert MTB/RIF for all patients, regardless of DR-TB risk.

**Total diagnostic costs include costs for all bacteriological TB and resistance tests and for CXR and/or antibiotic trial

**Table 4:** Incremental cohort and diagnostic costs per true TB case detected and treated, for 4 diagnostic strategies (Cost in US$2013).

	Total cohort costs	Total cohort diagnostic costs**	Total cohort treatment costs	True TB cases detected and initiated on treatment (TP)	Incremental True TB cases compared to baseline	Incremental total cohort costs compared to baseline	Incremental total diagnostic cost (US$2013) per incremental true TB case detected and initiated on treatment. Compared to:		Incremental total cohort cost (US$2013) per incremental True TB cases detected and initiated on treatment. Compared to:	
Diagnostic Strategy*	US$ 2013	US$ 2013	US$ 2013	n	n	US$ 2013	Baseline	Strategy 3†	Baseline	Strategy 3†
**1. Baseline: SSM. Clinical diagnosis. If prev treated, LPA if SSM+ or Culture/LPA/DST if SSM negative**.	7,173,573	619,042	6,554,530	10,188						
**2. Baseline: SSM. Clinical diagnosis. If prev treated, Xp to diagnose Rif Res if SSM+**	7,188,556	575,377	6,613,180	10,188	-	14,984	n.a.		n.a.	
**3. Xpert MTB/RIF for presumptive TB cases with prev TB history. SSM for new patients**.	8,509,863	720,523	7,789,340	11,016	828	1,336,290	123		1614	
**4. Xpert MTB/RIF for all patients**	10,492,396	1,639,643	8,852,753	13,380	3,192	3,318,823	320	389	1040	839

TP = true positive; DR-TB = drug-resistant TB, i.e. rifampicin-resistant; SSM = sputum smear microscopy

*Scenario 1: Perform SSM. If SSM positive and patient has been previously treated for TB, use LPA. If SSM negative and patient has been previously treated for TB, perform culture, LPA and/or DST.

Scenario 2: Perform SSM. If SSM positive and patient has been previously treated for TB, use Xpert in place of LPA. If SSM negative and patient has been previously treated for TB, perform culture, LPA and/or DST.

Scenario 3: Perform SSM only for patients not at DR-TB risk. Perform Xpert MTB/RIF for patient has been previously treated for TB.

Scenario 4: Perform Xpert MTB/RIF for all patients, regardless of DR-TB risk.

**Total diagnostic costs include costs for all bacteriological TB and resistance tests and for CXR and/or antibiotic trial

^†^ Strategy 3 being the next most effective option

### Population of 7.64 million

When applied to the whole country (Table 5), the total costs for diagnosis of TB and rifampicin resistance (not including treatment) among an estimated 7.64 million presumptive TB patients were highest for the Xpert-for-all scenario. Total diagnostic costs would comprise 19% of the annual TB control budget for SSM-only, 17% for the LPA replacement scenario, 22% for the SSM replacement scenario, and 50% for the Xpert-for-all scenario. The expanded Xpert deployment and usage across different scenarios, would lead to an increase in the case detection of TB, more significantly rifampicin resistant TB cases, which would warrant a substantial increase in budget for initiating the detected cases with appropriate treatment regimen. In the Xpert-for-all scenario the cost for 1^st^ line treatment would increase minimally compared to SSM-only. However, cost for 2nd line treatment would increase by 59%, resulting in an overall expected 22% increase in total treatment costs. Compared to the SSM-only scenario, the Xpert-for-all scenario is predicted to result in a $253 million increase in total national costs for TB diagnosis and treatment.

**Table 5:** Total diagnostic costs and treatment costs under the TB control program for 4 diagnostic strategies.

	Number of patients tested	Total diagnostic costs	Diagnostic costs as % of the budget**	Total number on TB treatment: TP+FP	1st line treatment	2nd line treatment	Total number on DR-TB treatment: TP+FP	Total treatment costs	Total costs
Diagnostic Strategy*	n [millions/year]	Million US$ 2013	(%)	n [millions/year]	Million US$ 2013	Million US$ 2013	n [millions/year]	Million US$ 2013	Million US$ 2013
**1. SSM Only**	7.64	$47.3	19%	1.19	$210	$291	0.050	$453	$548
**2. Xpert MTB/RIF as a replacement for LPA testing**	7.64	$43.9	17%	1.19	$210	$295	0.051	$461	$549
**3. Xpert MTB/RIF as a replacement for SSM for patients with previous TB history**	7.64	$55.0	22%	1.22	$213	$382	0.066	$540	$650
**4. Xpert MTB/RIF for all patients**	7.64	$125.2	50%	1.23	$212	$464	0.080	$552	$801

All costs are in millions, US$ 2013

TP = true positive; DR-TB = drug-resistant TB, i.e. rifampicin-resistant; FP = false positive; SSM = sputum smear microscopy

*Scenario 1: Perform SSM. If SSM positive and patient has been previously treated for TB, use LPA. If SSM negative and patient has been previously treated for TB, perform culture, LPA and/or DST.

Scenario 2: Perform SSM. If SSM positive and patient has been previously treated for TB, use Xpert in place of LPA. If SSM negative and patient has been previously treated for TB, perform culture, LPA and/or DST.

Scenario 3: Perform SSM only for patients not at DR-TB risk. Perform Xpert MTB/RIF for patient has been previously treated for TB.

Scenario 4: Perform Xpert MTB/RIF for all patients, regardless of DR-TB risk.

**The budget of the TB control program reported in the WHO 2014 report. (US$ 252 Million)

The mean diagnostic costs (for TB and rifampicin-resistant TB combined) per patient more than doubled for the Xpert-for-all scenario, from US$ 6.2 for SSM-only to US$ 16.4 (Table 6), representing a 102% increase in a mean diagnostic cost per true TB case initiated on treatment. The increase in mean total costs (including treatment) per true TB case initiated on treatment was about 11% from the baseline scenario to the Xpert-for-all scenario. Mean total costs per MDR case initiated on treatment were lowest in the Xpert-for-all scenario (US$ 11,099), and highest in scenario 2, with SSM as primary diagnostic tool and Xpert based DST, instead of LPA, for high risk TB cases (US$ 12,092).

**Table 6:** Mean costs per patient for 4 diagnostic strategies.

	Mean diagnostic cost^ǂ^			Mean treatment cost^ǂ^		Mean total cost^ǂ^		
Diagnostic Strategy*	per patient tested	per TP TB case initiated on treatment**	per TP MDR case detected	per TP TB case detected	per TP MDR case detected	per patient tested	per TP TB case detected	per TP MDR case detected
**1. SSM Only**	$6.2	$60.8	$999	$643	$10,579	$71.7	$704.1	$11,578
**2. Xpert MTB/RIF as a replacement for LPA testing**	$5.8	$56.5	$968	$649	$11,124	$71.9	$705.6	$12,092
**3. Xpert MTB/RIF as a replacement for SSM for patients with previous TB history**	$7.2	$65.4	$945	$707	$10,218	$85.1	$772.5	$11,163
**4. Xpert MTB/RIF for all patients**	$16.4	$122.5	$1,734	$662	$9,364	$104.9	$784.2	$11,099

TP = true positive; DR-TB = drug-resistant TB, i.e. rifampicin-resistant; FP = false positive; SSM = sputum smear microscopy

^**ǂ**^All costs in US$ 2013

*Scenario 1: Perform SSM. If SSM positive and patient has been previously treated for TB, use LPA. If SSM negative and patient has been previously treated for TB, perform culture, LPA and/or DST.

Scenario 2: Perform SSM. If SSM positive and patient has been previously treated for TB, use Xpert in place of LPA. If SSM negative and patient has been previously treated for TB, perform culture, LPA and/or DST.

Scenario 3: Perform SSM only for patients not at DR-TB risk. Perform Xpert MTB/RIF for patient has been previously treated for TB.

Scenario 4: Perform Xpert MTB/RIF for all patients, regardless of DR-TB risk.

**All diagnostic costs combined, for TB and DR-TB (16,492 true pulmonary TB cases among presumptive TB patients and 1,288 rifampicin resistant cases in the cohort).

**Fig 1:**
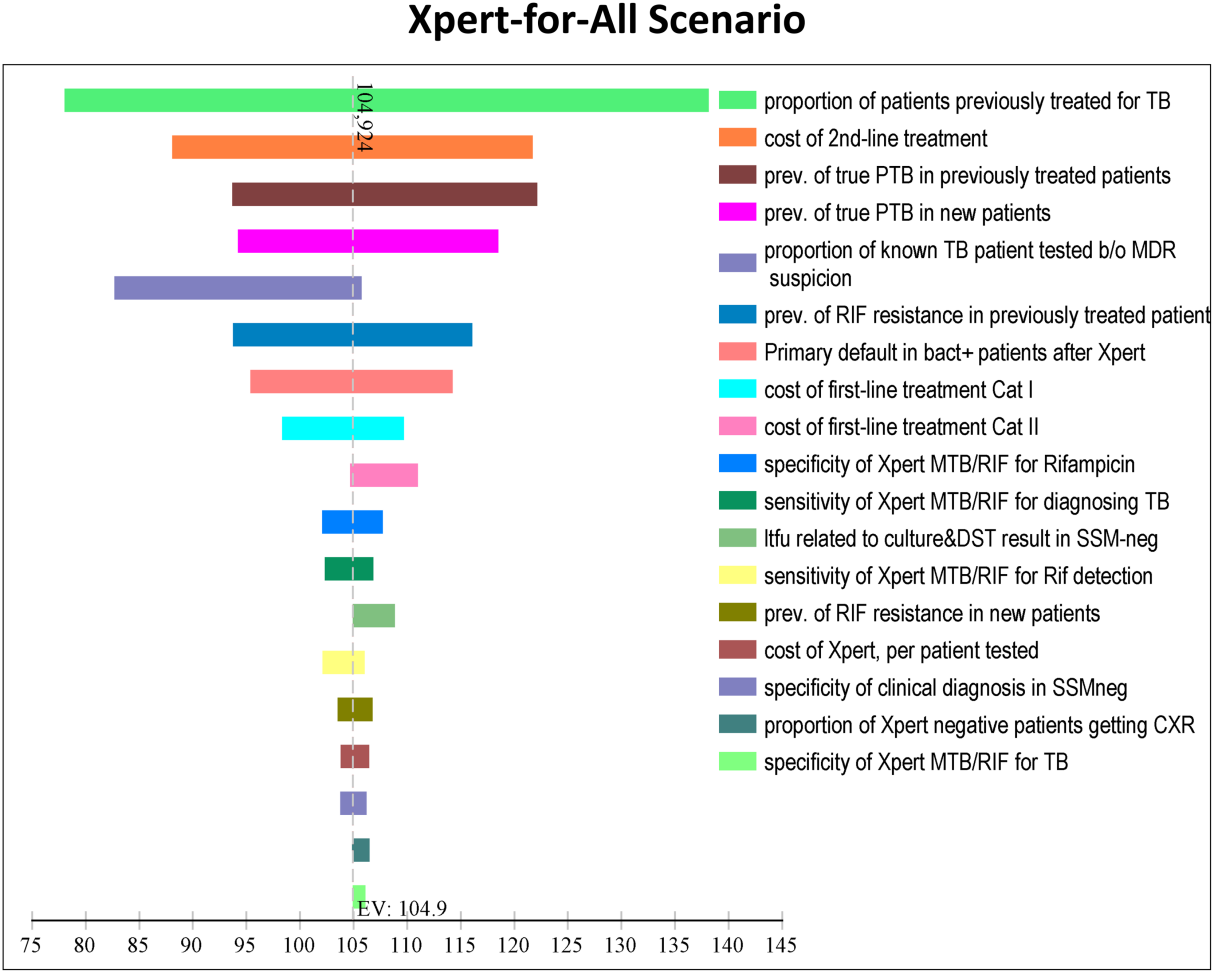
Tornado diagram showing the effect of variation in each parameter value on total costs (US$ per patient tested) for the Xpert for all scenario.

In Fig 1, the one-way effect of variation in model parameter values and assumptions on total cohort costs is shown for the top 18 variables (out of all included) in the Xpert-for all scenario. The largest variation in total costs was due to variation in parameters that increased or decreased the prevalence of TB in the cohort (in line with regional estimates of TB burden in India), and variation in parameters that affected second-line treatment cost.

### Use and interpretation of chest x-rays (CXRs)

If all presumptive TB patients with negative bacteriological tests (SSM or Xpert) received CXR, the need for TB treatment, both for a true positive and false positive TB diagnosis, would increase in all scenarios (Table A of S1 File). Total cohort costs would increase by approximately half in all scenarios, largely due to increased cost for first-line treatment. If CXR would be applied after negative SSM, but not after a negative Xpert result, TB case detection of true positive TB cases would be approximately the same in all scenarios. Diagnostic cost would still be highest in the Xpert-for-all scenario, but total cohort costs would be lowest in this scenario, as false positive cases would not be initiated on treatment. DR-TB case detection and associated costs of second-line treatment remained unchanged.

## Discussion

The WHO calls for the early diagnosis of TB, including universal DST and systematic screening of high-risk groups, as a critical component of the End TB Strategy. The RNTCP, seeking to control a growing TB epidemic while operating with a restricted budget, will need information regarding the associated costs and benefits to various diagnostic strategies in India to better inform Xpert scale-up and uptake and ultimately make progress towards the WHO End TB goals. This study found the upfront Xpert-for-all strategy to be associated with the greatest increase in TB and DR-TB case detection, and though it would have the highest associated total costs, the cost per true DR-TB case detected would be lower compared to the use of Xpert for DR-TB risk groups only.

The detection of true positive TB and DR-TB cases being highest for the upfront Xpert-for-all scenario is in line with previous findings [6, 7]. The Xpert for all strategy would increase the costs for diagnosis and treatment combined by approximately 46%, of which three quarters would be for increased treatment costs, especially for second-line treatment of additionally identified patients with DR-TB. Algorithms using Xpert only for patients at risk for MDR-TB would be more affordable, but less effective in increasing case detection. Also, the mean total cost per true DR-TB case detected and initiated on treatment would be lower with upfront Xpert-for-all compared to Xpert for DR-TB risk groups, only, primarily due to the Xpert-for-all identifying proportionally more DS-TB cases, which are cheaper to treat. The Xpert-for-all scenario was also the most robust to uncertainty in assumptions about the usage and performance of CXR in TB diagnosis. However, an expanded usage of Xpert across different strategies would require significant increased TB control budget to ensure that increased case detection is followed by appropriate care.

This study modeled the expected cost associated with roll-out of different Xpert scenarios to allow for the consideration of different diagnostic strategies for a set sample size given budget limitations. An earlier study demonstrated Xpert MTB/RIF to be a highly cost-effective as a replacement of SSM in India [4]. This was supported by the even lower average diagnostic costs per patient detected (and initiated on treatment) seen in this modeling study, which is largely explained by a lower per-patient test cost of the Xpert assay [15], and a higher prevalence of TB and drug-resistance TB in the current study under programmatic conditions. Our model predicted a 31% increase in detection of bacteriologically-confirmed pulmonary TB for the Xpert-for-all scenario, which was consistent with the Indian Xpert implementation study [7].

This study had a number of limitations. First, the analysis applied to patients in the public sector only. Diagnostics and treatment in the private sector were not included. Moreover, we assumed that patients made one TB diagnostic effort only, and so test-costs or cases detected at additional diagnostic visits were not considered [25]. Additionally, HIV-infected patients were not modeled as a separate group in this study. The HIV prevalence in India is low so the impact of this assumption on the overall result was expected to be small. Presumptive TB patients at increased risk of MDR were approximated by patients with a history of previous TB, comprising 17% of the cohort. Other patients at risk for DR-TB, such as MDR-contacts, were also not modeled as a separate group, as their number was expected to be small. Additionally, it should be noted that varying the assumptions about the prevalence of drug resistance in general showed a large effect on the expected total cohort costs, implying that the estimation of costs for certain regions may vary considerably along with regional variations in the distribution of drug resistance [6].

This study took the perspective of a TB service-provider. Therefore, costs encountered by the patients themselves, whether direct or indirect, were not considered in this analysis. Notably, patient pathways to TB diagnosis are complex and often include providers other than, or in addition to, those encountered in the public sector [25]. However, a diagnostic test such as Xpert, with higher sensitivity, would be expected to reduce the number of TB patient visits, especially given a reduction in the number of additional visits for CXR and/or repeat testing after negative test results were obtained. Practices related to clinical diagnosis and the use of CXR, as well as variation in SSM sensitivity (reflecting smear method and quality variations) may also have a substantial effect on costs, including treatment costs, even if the patients themselves paid the cost of CXR.

The uptake of rapid TB and DR-TB diagnostics such as Xpert MTB/RIF has become an RNTCP implementation priority as India works to control a growing TB epidemic. Economic modeling of the TB epidemic in India and different diagnostic strategies found the Xpert-for-all strategy to be the most costly, though it was the most effective in improving case detection and had the lowest mean total costs per DR-TB case initiated on treatment. Given current budget limitations, the RNTCP will have to consider these findings in the context of other TB control activities to determine the best approach to Xpert scale-up for TB and DR-TB control in India.

## Supporting information

S1 File SUPPLEMENT to Scaling-up the Xpert MTB/RIF assay for the diagnosis of tuberculosis and rifampicin resistant tuberculosis in India: An economic analysis. (DOCX)
