# Supplementary material for: Scaling-up the Xpert MTB/RIF assay for the detection of tuberculosis and rifampicin resistance in India: An economic analysis
Source: PLoS One. 2017 Sep 7;12(9):e0184270. doi: 10.1371/journal.pone.0184270 (PMC5589184; doi:10.1371/journal.pone.0184270)
Supplement: S1 File — (DOCX) [file pone.0184270.s001.docx]

**SUPPLEMENT to**

**Scaling-up the Xpert MTB/RIF assay for the diagnosis of tuberculosis and rifampicin resistant tuberculosis in India: An economic analysis**

**METHODS**

Additional information on:

**Epidemiological characteristics of simulated cohort**

To estimate the prevalence of truly bacteriologically positive (culture positive) PTB among the cohort, we first calculated a prevalence of truly bacteriologically positive (culture positive) PTB among the cohort was calculated based on the sensitivity and specificity of Xpert and SSM as reported in the literature {Steingart, 2006 364 /id} {Steingart, 2014 5114 /id}, and the proportion of Xpert-positive and SSM-positive PTB cases in each respective arm of the implementation study {Sachdeva, 2014 5137 /id}, as follows: estimated true prevalence=((proportion PTB cases identified-(1-specificity))/sensitivity. The average of the two phases provides a prevalence point estimate of 19.1% in new patients and 34.1% in patients previously treated for TB. These values were then divided by 1.27 to adjust to RNCTP national data, estimating the prevalence in new patients is to be 15%.

**Additional assumptions on diagnostic scenarios**

*Baseline (SMM-Only)*

- In false positive SSM+ PTB cases we assumed that the LPA gives a negative result (not MTB), meaning that false positive PTB cases cannot have an MDR diagnosis on top.

*Xpert MTB/RIF for all*

- The proportion of patients subjected to the clinical diagnostic process after a negative Xpert was half of that after SSM in primary analysis. This assumption was based on observations in the implementation study {Sachdeva, 2014 5137 /id} showing that 8% of all PTB cases diagnosed in the intervention phase were clinically diagnosed and 16% in the baseline.
- In absence of data on the actual numbers of Xpert-negative presumptive TB patients that were further evaluated with X-ray we make assumptions that fit with the observed number of clinically diagnosed cases in the implementation study: In the primary analysis, we assumed that the proportion of patients subjected to the clinical diagnostic process after a negative Xpert was half of that after SSM, and the false positive fraction (1-specificity) was also half.
- LPA for the detection of rifampicin resistance {Bwanga, 2009 5128 /id} performed similarly in smear-positive patients, for whom LPA is directly done on clinical sample, as in smear-negative culture positive patients for whom LPA done on isolate obtained from culture {Ling, 2008 472 /id}.

**Treatment costs**

Costs for a health facility visit were taken from WHO-CHOICE {World Health Organization, 2012 473 /id}. We assume that visits were to the lowest level (health centre, no beds). For Xpert, which is not as decentralized as SSM a proportion (approximately 50%) will get an additional cost for sputum transportation.

We assumed the costs of one full course of treatment for all patients who started TB treatment. Additional (prolonged) treatment for e.g. default was not considered.

**Sensitivity analysis**

List of deterministic sensitivity analyses

- Epidemiological parameters (prevalence of TB and rifampicin resistance)
- Assumptions about test accuracy
- Assumptions about clinical diagnosis
  - proportion of patients examined by CXR,
  - the accuracy of clinical diagnosis/CXR
  - a scenario in which no clinical diagnosis is done after a negative Xpert test (as recommended in the new guideline)
- Cost for diagnostic tests and treatment
- Addition of sputum transportation to centralized Xpert sites

**Treatment costs**

An example of how treatment costs were calculated is shown below:

**Table A.** Effect of including chest x-rays (CXRs) in 4 different diagnostic scenarios

| **Diagnostic Strategy*** | **True TB cases detected and initiated on treatment (TP)** | | **False TB cases detected and initiated on treatment (FP)** | **Total diagnostic costs†** | **Total number on TB treatment (TP+FP)** | **1st line treatment costs** | **Total number on DR-TB treatment (TP+FP)** | **2nd line treatment costs** | **Total treatment costs** | **Total costs** |  |
| --- | --- | --- | --- | --- | --- | --- | --- | --- | --- | --- | --- |
| **PRIMARY ANALYSIS** | **n** | **(%**)** | **n** | **US$ 2013** | **n** | **US$ 2013** | **n** | **US$ 2013** | **US$ 2013** | **US$ 2013** |  |
| 1. SSM Only | 10,188 | 62% | 5,365 | 619,042 | 15,553 | 2,747,408 | 655 | 3,807,122 | 6,554,530 | 7,173,573 |  |
| 2. Xpert MTB/RIF as a replacement for LPA testing | 10,188 | 62% | 5,365 | 575,377 | 15,553 | 2,745,435 | 665 | 3,867,745 | 6,613,180 | 7,188,556 |  |
| 3. Xpert MTB/RIF as a replacement for SSM for patients with previous TB history | 11,016 | 67% | 4,969 | 720,523 | 15,985 | 2,782,695 | 861 | 5,006,646 | 7,789,340 | 8,509,863 |  |
| 4. Xpert MTB/RIF for all patients | 13,380 | 81% | 2,697 | 1,639,643 | 16,076 | 2,771,662 | 1,046 | 6,081,091 | 8,582,753 | 10,492,396 |  |
| **ALTERNATIVE 1: all SSM neg and Xpert neg patients receive CXR (implying higher sensitivity and lower specificity)** | | | | | | | | | | | |
| 1. SSM Only | 13,083 | 79% | 26,098 | 949,806 | 39,181 | 6,403,161 | 655 | 3,807,122 | 10,210,283 | 11,160,089 |  |
| 2. Xpert MTB/RIF as a replacement for LPA testing | 13,083 | 79% | 26,098 | 906,141 | 39,181 | 6,401,187 | 665 | 3,867,745 | 10,268,932 | 11,175,073 |  |
| 3. Xpert MTB/RIF as a replacement for SSM for patients with previous TB history | 13,400 | 81% | 26,026 | 1,047,902 | 39,426 | 6,402,916 | 861 | 5,006,646 | 11,409,562 | 12,457,464 |  |
| 4. Xpert MTB/RIF for all patients | 14,341 | 87% | 25,608 | 1,961,390 | 39,949 | 6,455,923 | 1,046 | 6,081,091 | 12,537,013 | 14,498,403 |  |
| **ALTERNATIVE 2: all SSM neg receive CXR (implying higher sensitivity and lower specificity) but Xpert neg patients do not** | | | | | | | | | | | |
| 1. SSM Only | 13,083 | 79% | 26,098 | 949,806 | 39,181 | 6,403,161 | 655 | 3,807,122 | 10,210,283 | 11,160,089 |  |
| 2. Xpert MTB/RIF as a replacement for LPA testing | 13,083 | 79% | 26,098 | 906,141 | 39,181 | 6,401,187 | 665 | 3,867,745 | 10,268,932 | 11,175,073 |  |
| 3. Xpert MTB/RIF as a replacement for SSM for patients with previous TB history | 13,108 | 79% | 22,337 | 950,776 | 35,444 | 5,649,645 | 861 | 5,006,646 | 10,656,291 | 11,607,067 |  |
| 4. Xpert MTB/RIF for all patients | 13,235 | 80% | 731 | 1,317,488 | 13,965 | 2,445,549 | 1,046 | 6,081,091 | 8,526,639 | 9,844,127 |  |

All costs in US$ 2013

TP= true positive; DR-TB= drug-resistant TB, i.e. rifampicin-resistant; FP= false positive; SSM= sputum smear microscopy

*Scenario 1: Perform SSM. If SSM positive and patient has been previously treated for TB, use LPA. If SSM negative and patient has been previously treated for TB, perform culture, LPA and/or DST.

Scenario 2: Perform SSM. If SSM positive and patient has been previously treated for TB, use Xpert in place of LPA. If SSM negative and patient has been previously treated for TB, perform culture, LPA and/or DST.

Scenario 3: Perform SSM only for patients not at DR-TB risk. Perform Xpert MTB/RIF for patient has been previously treated for TB.

Scenario 4: Perform Xpert MTB/RIF for all patients, regardless of DR-TB risk.

** Out of 16,492 true pulmonary TB cases among presumptive TB patients and 1,288 rifampicin resistant cases in the cohort

^†^Total diagnostic costs include costs for all bacteriological TB and resistance tests and for CXR and/or antibiotic tria

**Figure A.** Existing algorithm for pulmonary TB under the current programme.

**Figure B.**
